# Integrative analysis of EMT-driving genes identifies a prognostic signature and GJB2 as a potential biomarker in glioblastoma

**DOI:** 10.3389/fcell.2025.1754988

**Published:** 2026-01-14

**Authors:** Xiao-fei Liu, Bing Wang, Min Zhou, Duo Gong, Jia-hui Zhang, Jun-yan He, Wen-tao Xiao

**Affiliations:** 1 Department of Neurosurgery, The Second Affiliated Hospital, Hengyang Medical School, University of South China, Hengyang, China; 2 Key Laboratory for Arteriosclerology of Hunan Province, Hengyang Medical School, Institute of Cardiovascular Disease, University of South China, Hengyang, China; 3 Health Check Center, The Second Affiliated Hospital, Hengyang Medical School, University of South China, Hengyang, China; 4 Department of Oncology, The First Affiliated Hospital, Hengyang Medical School, University of South China, Hengyang, China; 5 Department of Radiation Oncology, Affiliated Hospital of Nantong University, Nantong, China

**Keywords:** EMT-driving genes, epithelial-mesenchymal transition, GJB2, glioblastoma, prognostic signature, tumor immune microenvironment

## Abstract

**Background:**

Glioblastoma (GBM) is an aggressive malignancy of the central nervous system characterized by rapid progression, therapeutic resistance, and poor prognosis. Epithelial–mesenchymal transition (EMT) contributes to tumor plasticity and immune remodeling in GBM. Identification of EMT-driving genes (EDGs) with prognostic and therapeutic relevance may provide insights into disease progression and precision management. This study aimed to systematically identify prognostic EDGs and to construct a robust EMT-based prognostic model for GBM.

**Methods:**

Univariate Cox regression analyses were performed across five GBM cohorts (TCGA, CGGA-693, CGGA-325, GSE83300, and GSE74187) to rank genes according to hazard ratios, followed by Gene Set Enrichment Analysis (GSEA), which identified EMT as the pathway most strongly associated with adverse survival. EMT-related genes from MSigDB and dbEMT2.0 were intersected with TCGA-derived prognostic genes to obtain 145 EDGs. An EMT-related prognostic signature was generated using LASSO Cox regression and validated in independent datasets. Functional analyses, including Gene Ontology (GO), Kyoto Encyclopedia of Genes and Genomes (KEGG), weighted gene co-expression network analysis (WGCNA), and GSEA, elucidated biological processes associated with the risk signature. Immune infiltration analyses (ESTIMATE, CIBERSORT) and drug sensitivity analyses characterized the tumor microenvironment and therapeutic response. Random Forest analysis and *in vitro* assays identified and validated GJB2 as a key mediator of GBM progression.

**Results:**

A total of 145 EDGs were identified by integrating survival-associated genes from TCGA with EMT-related genes from MSigDB and dbEMT2.0. An EMT-related prognostic signature was developed and validated across five GBM cohorts. The risk score stratified patients into high- and low-risk groups with significantly different survival outcomes and remained an independent prognostic factor in multivariate Cox analyses. A nomogram integrating the risk score with age, IDH mutation status, and MGMT promoter methylation demonstrated strong predictive performance. Immune profiling revealed that the high-risk group exhibited an “immune-inflamed yet immunosuppressed” phenotype characterized by elevated macrophage and regulatory T-cell infiltration. Drug sensitivity analyses suggested that high-risk GBM may respond better to paclitaxel and tamoxifen. Random Forest modeling and *in vitro* experiments identified GJB2 as an oncogenic driver that promotes GBM cell proliferation and migration.

**Conclusion:**

Our findings provide a clinically applicable EMT-based prognostic framework that links transcriptional plasticity to patient outcomes in GBM and identify GJB2 as a promising therapeutic target.

## Introduction

Glioma is the most prevalent and aggressive primary tumor of the central nervous system, noted for its rapid progression, elevated rates of recurrence, and unfavorable prognosis (Tan et al., 2020). Glioblastoma (GBM), classified as grade IV glioma, represents the most malignant subtype and accounts for the majority of glioma-related mortality worldwide (Ostrom et al., 2023). The current standard of care for newly diagnosed GBM is an aggressive multimodal treatment strategy comprising maximal safe surgical resection, followed by radiotherapy with concurrent and adjuvant temozolomide chemotherapy, a regimen commonly referred to as the Stupp protocol (Stupp et al., 2005). However, despite this intensive treatment, the prognosis for patients with GBM remains poor. The primary challenges contributing to this dismal outcome are nearly universal tumor recurrence and profound therapeutic resistance, which limit the median overall survival to a 15–18 months and the 5-year survival rate to under 10% (Thakkar et al., 2014; Lan et al., 2025). To address this challenge, research has increasingly focused on the complex biological processes that drive GBM’s aggressiveness. Accumulating evidence suggests that glioma progression is not solely driven by static genetic alterations but also by phenotypic plasticity and dynamic remodeling within the tumor microenvironment (TME), which collectively facilitate tumor cell invasion, immune evasion, and treatment resistance (Gargini et al., 2020; Sharma et al., 2023).

Among the molecular processes contributing to glioma malignancy, Epithelial-mesenchymal transition (EMT) plays a pivotal role. EMT is a fundamental biological process wherein polarized epithelial cells lose key cell-cell adhesion molecules and acquire a migratory, invasive mesenchymal phenotype, characterized by expression of markers such as N-cadherin and vimentin (Kalluri and Weinberg, 2009). In epithelial carcinomas, EMT activation is a well-established driver of tumor progression, endowing cancer cells with the capacity for invasion and metastasis, the acquisition of cancer stem cell (CSC) properties, and profound therapeutic resistance (Nieto et al., 2016). Although glioblastoma originates from neuroepithelial tissue and lacks a true epithelial component, it undergoes a highly analogous process, often termed a mesenchymal transition (MT) or EMT-like program (Iwadate, 2016; Segerman et al., 2016). The mesenchymal glioblastoma subtype, distinguished by EMR2 upregulation, is associated with increased invasiveness and poor patient survival (Buzatu et al., 2024). This transition is driven by multiple oncogenic and stress-related signaling pathways, including TGF-β, STAT3, and NF-κB, which collectively confer survival advantages and resistance to chemoradiotherapy (Bhat et al., 2013; Lau et al., 2015). Moreover, it sustains glioma stem cell (GSC) plasticity, intratumoral heterogeneity, and immune evasion, thereby fostering aggressive tumor behavior and recurrence (Yabo et al., 2022; Luo et al., 2023). Taken together, EMT-associated pathways and transcriptional reprogramming represent key mechanisms driving glioma progression and therapy resistance, underscoring the importance of EMT-related genes for prognostic prediction and patient stratification.

In recent years, numerous prognostic signatures based on immune-, metabolic-, and ferroptosis-related gene sets have been proposed to predict outcomes in GBM (Zhuo et al., 2020; Lei et al., 2021; Tian et al., 2022; Ye et al., 2022; Tu et al., 2025). Specifically, several studies have also recognized the importance of EMT and have developed prognostic models based on EMT-related gene sets at the pan-glioma level (Luo et al., 2022; Fu et al., 2024; Zheng et al., 2024). However, many of the existing EMT-related models face significant limitations, including small sample sizes derived from single cohorts, lack of validation across diverse independent datasets, and insufficient integration with key clinical prognostic factors such as age, IDH mutation status, and MGMT promoter methylation, which remain the cornerstones of GBM risk stratification (Takashima et al., 2019; Wu et al., 2022; Xiao et al., 2023). Consequently, a distinct and pressing requirement persists for the creation and validation of a more comprehensive and robust prognostic signature that incorporates an innovative, systematically identified EMT-related genes with these essential clinical variables to improve the accuracy and clinical applicability of outcome prediction for GBM patients.

In this study, we developed and validated a clinically relevant prognostic signature for GBM based on EMT-related genes. Based on univariate Cox regression analyses across five independent GBM datasets, followed by gene set enrichment analysis (GSEA), we identified EMT as one of the most significantly enriched pathways associated with poor overall survival. These findings suggested that EMT activation might represent a key biological process underlying GBM aggressiveness and patient prognosis. Guided by this finding, we next sought to construct an EMT-associated prognostic signature. Specifically, we systematically identified a set of prognostic EMT-driving genes (EDGs) by integrating univariate Cox regression results from multiple independent glioblastoma cohorts available in the TCGA, CGGA, and GEO databases with established EMT gene sets from the MSigDB and dbEMT2.0 databases. Using these EDGs, we developed a prognostic signature through LASSO-Cox regression, which demonstrated high predictive accuracy and was successfully validated in multiple independent datasets. Among the genes constituting our signature, Random Forest analysis identified GJB2 as the most pivotal gene for predicting prognosis. We then experimentally validated that knockdown of GJB2 significantly inhibited the proliferation, migration, and invasion of GBM cells *in vitro*. Collectively, our study not only provides a robust, clinically integrated EMT-based signature for improved risk stratification in GBM but also identifies and validates GJB2 as a key driver and potential therapeutic target within this malignant process.

## Materials and methods

### Data acquisition

**FIGURE 1:**
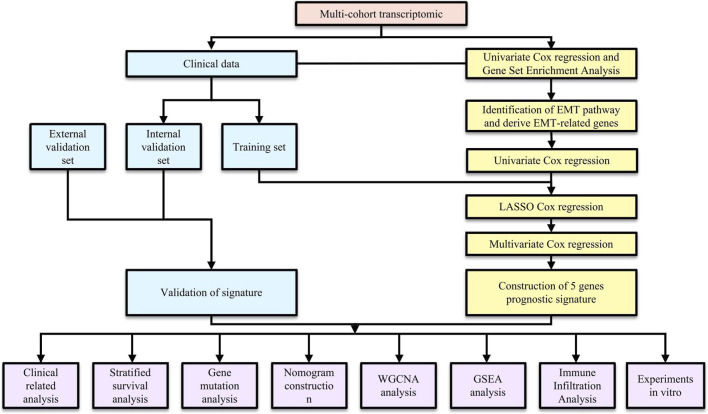
The flowchart of the whole study.

Patient data from publicly available glioblastoma (GBM) cohorts were collected from The Cancer Genome Atlas (TCGA, https://portal.gdc.cancer.gov/), Gene Expression Omnibus (GEO, https://www.ncbi.nlm.nih.gov/geo/), and the Chinese Glioma Genome Atlas (CGGA, https://www.cgga.org.cn/). After excluding samples without available survival information or with an overall survival time of ≤30 days, 471 GBM samples were included in the final analysis. The TCGA-GBM dataset (n = 146) was used as the discovery cohort for model development and internal validation. For external validation, datasets from GEO (GSE83300, n = 50; GSE74187, n = 59) and CGGA (CGGA-693, n = 132; CGGA-325, n = 84) were utilized to external validation to assess model robustness and generalizability. Genes with a median raw count of 0 across samples were removed prior to DESeq2 normalization and variance-stabilizing transformation. All datasets were preprocessed using log2 transformation and normalization. For count-based RNA-seq data, normalization was performed with DESeq2. (https://bioconductor.org/packages/DESeq2/). This approach was adopted to ensure comparability across datasets. The overall workflow of the study is visually represented in Figure 1.

### Identification of prognosis-associated pathways by GSEA

Following the exclusion of samples lacking clinical information, we performed univariate Cox regression analysis within each cohort to evaluate the prognostic relevance of each gene. Log2-transformed hazard ratios (log2HRs) were used to quantify each gene’s association with survival, thereby distinguishing putative risk-associated from protective genes. Genes were subsequently ranked according to their log_2_HR values for Gene Set Enrichment Analysis (GSEA), which was performed to identify hallmark biological pathways associated with patient survival. The hallmark gene sets were downloaded from the Molecular Signatures Database (MSigDB, accessed in March 2025).

### Identification of EMT-driving genes

Based on the GSEA results, HALLMARK_EPITHELIAL_MESENCHYMAL_TRANSITION (EMT) was identified as one of the top pathways influencing the survival of GBM patients. Based on this finding, we collected all currently known EMT-related genes through literature review and database search. A total of 1,317 EMT-associated genes were obtained from the MSigDB (accessed in March 2025) and the dbEMT2.0 database (http://dbemt.bioinfo-minzhao.org/, accessed in March 2025). This combined strategy has been adopted in recent transcriptome-based prognostic studies to ensure both biological relevance and adequate pathway coverage (Li et al., 2024). In the TCGA-GBM cohort, prognostic genes that exhibited a significant correlation with overall survival (P < 0.05) were identified. Univariate Cox analysis with a nominal P < 0.05 threshold was used as an initial feature-reduction step prior to LASSO Cox regression and multivariate modeling. The resulting prognostic genes were then intersected with EMT-related genes obtained from MSigDB and dbEMT2.0, resulting in 145 EMT-driving genes (EDGs) for subsequent analysis.

### Construction and validation of a prognostic signature based on EDGs

The TCGA cohort was randomly split into training and testing sets at a 1:1 ratio. No stratification by clinical covariates was applied at the data-splitting stage. Univariate Cox proportional hazards models were first applied to screen EMT-driving genes (EDGs) associated with overall survival in the training set. To further refine prognostic candidates, LASSO-penalized Cox regression was implemented using the glmnet R package. Gene expression predictors were automatically standardized (standardize = TRUE), and the optimal penalty parameter (λ) was determined by 10-fold cross-validation using the cv. glmnet function. The λ value corresponding to the minimum cross-validated partial likelihood deviance (lambda.min) was selected. Genes retained at this λ were subsequently entered into a multivariable Cox regression model to identify independent prognostic genes. To compute the risk score for each individual patient, we employed a specific formula: Risk Score = (exp gene1 × coef1) + (exp gene2 × coef2) + … + (exp gene n × coefn). In this equation, “exp” denotes the mRNA expression levels of the respective genes, while “coef” signifies the regression coefficients obtained from the multivariate Cox regression analysis. Utilizing the calculated median risk score, we classified glioblastoma (GBM) patients into two categories: high-risk and low-risk groups. Subsequently, we conducted Kaplan-Meier survival analysis to compare the survival rates between these two classifications. The statistical significance of the observed differences was assessed through the log-rank test. Furthermore, we executed Time-dependent receiver operating characteristic (ROC) curve analysis using the pROC R package (Robin et al., 2011), concentrating on survival outcomes at 1-year, 2-year, and 3-year intervals to evaluate the predictive capability of our gene signature. The area under the ROC curve (AUC), along with sensitivity and specificity metrics, were utilized to determine predictive accuracy. To validate the robustness and reliability of the expression-derived gene signature (EDGs), we performed independent validation internally in the TCGA test set and the full TCGA cohort as well as four external datasets (GSE83300, GSE74187, CGGA-693, and CGGA-325). This methodology was designed to ascertain the generalizability and consistency of our results across diverse datasets.

### Association between EDGs signature and clinicopathological characteristics

The associations between the EDG-based risk signature and clinicopathological characteristics were depicted utilizing the R packages ggplot2 (https://CRAN.R-project.org/package=ggplot2), ggpubr (https://CRAN.R-project.org/package=ggpubr), and pheatmap (https://CRAN.R-project.org/package=pheatmap). Stratified survival analyses were implemented with the survival (https://CRAN.R-project.org/package=survival) and survminer (https://CRAN.R-project.org/package=survminer) packages to assess the prognostic relevance of the signature within different subgroups. Additionally, both univariate and multivariate Cox regression analyses were carried out using the survival package to determine whether the risk score served as an independent prognostic factor.

### Genomic alterations and tumor mutational burden

The evaluation of tumor mutational burden (TMB) and mutation profiles across high-risk and low-risk cohorts was conducted utilizing R packages such as maftools (Mayakonda et al., 2018), ggplot2, and forestplot (accessible at https://CRAN.R-project.org/package=forestplot).

### Nomogram construction

The prognostic nomogram was created utilizing the rms R package (available at https://CRAN.R-project.org/package=rms) and included prognostic variables such as age, IDH status, MGMT promoter methylation status, and risk score. To validate the nomogram’s reliability, calibration curves were used to examine the relationship between the predicted probabilities and actual outcomes, thereby assessing predictive accuracy. An additional evaluation of the predictive efficacy was conducted by calculating the concordance index (C-index) and time-dependent AUCs. This analysis was performed utilizing the R packages riskRegression (accessible at https://CRAN.R-project.org/package=riskRegression) and timeROC (Blanche et al., 2013), facilitating a quantitative appraisal of the model’s discriminatory power.

### WGCNA

A weighted gene co-expression network was constructed utilizing the WGCNA R package (Langfelder and Horvath, 2008) to identify gene modules associated with the EDG-based risk score. Within the TCGA-GBM dataset, soft-thresholding power of 12 was applied to maintain the scale-free topology of the network. Network construction and module detection were performed using the blockwiseModules function with a minimum module size of 100 genes, and modules with highly similar expression profiles were merged using a cut height of 0.25 (mergeCutHeight = 0.25). Genes exhibiting similar expression profiles were organized into separate modules, with the module demonstrating the strongest correlation to the EDGs signature being chosen for further functional enrichment analysis.

### Functional enrichment analyses

Analyses for functional enrichment, which encompassed Gene Ontology (GO), Kyoto Encyclopedia of Genes and Genomes (KEGG), and Hallmark pathway evaluations, were conducted utilizing the R packages clusterProfiler, org. Hs.eg.db (https://bioconductor.org/packages/org.Hs.eg.db/), and enrichplot (https://bioconductor.org/packages/enrichplot/).

### GSEA

We performed Gene Set Enrichment Analysis (GSEA) to assess hallmark pathways that showed significant enrichment in the high-risk and low-risk groups. Genes were ordered based on their differential expression between these cohorts, and pathway enrichment analysis was carried out using the “clusterProfiler” R package (Xu et al., 2024). Pathways with adjusted p-values less than 0.05 were deemed significantly enriched.

### Immune infiltration analysis

The ESTIMATE R package (Yoshihara et al., 2013) was utilized to evaluate the immune infiltration and the tumor immune microenvironment by calculating immune scores, stromal scores, and composite scores from ESTIMATE. Additionally, the GSVA R package (Hänzelmann et al., 2013) was used to perform single-sample gene set enrichment analysis (ssGSEA) to measure the infiltration levels of 28 subsets of tumor-infiltrating immune cells. To assess the relationship between immune cell infiltration levels and risk scores, Spearman correlation analyses were employed.

### Drug sensitivity analysis

To identify potential therapeutic agents associated with the EMT-based risk signature, we turned to the CellMiner database (http://discover.nci.nih.gov/cellminer/) (Reinhold et al., 2012) to analyze the association between the risk score and drug sensitivity. Given the limited number of glioblastoma-derived cell lines in the NCI-60 panel, this analysis was designed as an exploratory, hypothesis-generating study. Further experimental validation in GBM-relevant cellular models will be required to assess the translational relevance of the identified associations. The analysis covered 216 drugs that have received FDA approval and 574 compounds being tested in clinical trials. Due to the limited number of glioblastoma-derived lines, a pan-cancer framework was adopted. For each cell line, the risk score was computed from the expression of the five signature genes, and lines were classified as high- or low-risk based on the median score. In the NCI-60 drug response analysis, cell lines/drugs with >60% missing values were excluded to reduce instability. Pearson correlation was used to evaluate the relationship between IC50 values and risk scores, while Wilcoxon rank-sum tests compared drug sensitivities between subgroups. Cell lines with >60% missing data were excluded, and all plots were generated using ggplot2 in R. These analyses were based on cell-line data and did not incorporate data obtained from patients.

### Cell culture

The human glioblastoma cell lines U251 and U87 were obtained from HyCyte™ (China). Both cell lines were cultured in DMEM (PM150210, Procell, China), supplemented with 10% fetal bovine serum (164210, Procell, China) and 1% penicillin-streptomycin solution (PB180120, Procell, China). All cells were grown at 37 °C in a humidified atmosphere with 5% CO_2_.

### GJB2 knockdown

To silence GJB2, custom-synthesized small hairpin RNAs (shRNAs) sequences (Sangon Biotech (Shanghai) Co., Ltd.) targeting GJB2 and cloned into the pLKO.1 vector (Addgene, Watertown, MA, United States). Primer sequences are shGJB2-F: 5′- CCG​GGT​CTT​CAC​AGT​GTT​CAT​GAT​TCT​CGA​GAA​TCA​TGA​ACA​CTG​TGA​AGA​CTT​TTT​G-3′, shGJB2-R: 5′-AAT​TCA​AAA​AGT​CTT​CAC​AGT​GTT​CAT​GAT​TCT​CGA​GAA​TCA​TGA​ACA​CTG​TGA​AGA​C-3′. Lentiviral packaging was conducted in 293T cells utilizing second-generation packaging plasmids, specifically psPAX2 and pMD2. G (Addgene). U251 and U87 cells were infected with either control (shNC) or GJB2-targeting (shGJB2) lentivirus for 48 h, followed by puromycin selection to establish stable knockdown cell lines.

### Western blot

The Western blot procedure followed methods reported in earlier studies (Fan et al., 2023; Liu et al., 2025) with minor modifications. Cells were lysed in RIPA buffer supplemented with a protease inhibitor cocktail to extract total protein, and total protein concentrations were quantified using the BCA Protein Assay Kit (Abiowell, AWB0104b, China). Equal quantities of protein were separated using SDS-PAGE and subsequently transferred onto Hybond ECL membranes (GE Healthcare, Chicago, IL, United States). After blocking, membranes were incubated overnight at 4 °C with GJB2 (Proteintech,1:1000 16960-1-AP, China), E-cadherin (Sellect, 1:1000, TA0131, China), N-cadherin (Abmart, 1:1000, T55015, China) and anti-Tubulin (Abiowell, 1:10000, AWA80025, China) primary antibodies, followed by HRP-conjugated secondary antibodies. Signals were visualized using ECL (Suzhou Xinsaimei Biotechnology Co., Ltd., P10100, China). Each assay was independently repeated in triplicate to ensure the reliability of the results. Each assay was independently repeated in triplicate to ensure the reliability of the results.

### Cell proliferation assay

Cell proliferation was evaluated using the CCK8 assay (Abiowell, AWC0114, China). U251 and U87 cells transfected with either shNC or shGJB2 were resuspended in complete culture medium and seeded into 96-well plates (1 × 10^3 cells/well). At the indicated time points, 10 μL of CCK-8 solution was added to each well, and the plates were incubated for 1.5 h at 37 °C under standard culture conditions. The absorbance was recorded at 450 nm using a microplate reader to determine cell viability. Each experiment was performed with five biological replicates (n = 5) to ensure reproducibility and statistical reliability.

### Wound healing assay

A wound-healing assay was conducted to assess the migratory capacity of glioma cells. U251 and U87 cells stably expressing shNC or shGJB2 were seeded in six-well plates at a density of 5 × 10^4^ cells/mL and cultured until a confluent monolayer formed. A linear scratch was created across the cellular monolayer utilizing a sterile 200 μL pipette tip, followed by the elimination of cellular debris through rinsing with PBS. The cells were then cultured in serum-free medium under standard conditions. Images were captured at 0 h and 24 h after scratching using a phase-contrast microscope at ×10 magnification. The wound closure was analyzed with ImageJ software, and the migration rate was expressed as: Wound healing rate = (0 h area - 24 h area)/(0 h area) × 100%.

### Transwell assay

Cell invasion was assessed using a Transwell chamber assay (BIOFIL, China). U251 and U87 cells stably expressing shNC or shGJB2 were suspended in serum-free DMEM and seeded into the upper chambers at a density of 1 × 10^3^ cells per 100 μL. The lower chamber was filled with 600 μL of DMEM containing 10% FBS as a chemoattractant. Following incubation for 12 h at 37 °C in a 5% CO_2_ atmosphere, cells that remained on the upper surface were carefully removed, while those that had migrated to the lower surface were fixed with 4% paraformaldehyde for 15 min and stained with 0.1% crystal violet for 20 min. Cell counts were performed in five randomly selected fields, and ImageJ software was used for quantitative analysis. All assays were repeated in triplicate. The Number of invading cells were quantified in ImageJ.

### Statistical analysis

Statistical analyses were performed using R software (version 4.2.2). Key packages included DESeq2 (v1.48.1), glmnet (v4.1-10), pROC (v1.19.0.1), clusterProfiler (v4.16.0), GSVA (v2.2.0), estimate (v1.0.13), WGCNA (v1.73), survival (v3.8-3), survminer (v0.5.0), maftools (v2.24.0), rms (v8.0-0), riskRegression (v2025.5.20), and timeROC (v0.4). Experimental data, including qPCR, CCK-8, colony formation, and migration assays, were analyzed using GraphPad Prism (version 9.5). Each experiment included at least three independent biological replicates. Two-group comparisons were performed using two-tailed Student’s t-tests, and significance was defined as P < 0.05 (*P < 0.05; **P < 0.01; ***P < 0.001; ****P < 0.0001). Univariate Cox regression models were employed to evaluate the prognostic implications of EMT-driving genes, and multiple testing correction was applied using the Benjamini-Hochberg false discovery rate (FDR) method. Genes with FDR-adjusted P < 0.05 were considered significant. Correlations between risk scores and immune cell infiltration were calculated using Spearman’s rank correlation. FDR correction was also applied to correlation analyses, with adjusted P < 0.05 regarded as significant.

## Results

### Identification of EDGs based on GSEA and cox regression analysis

**FIGURE 2:**
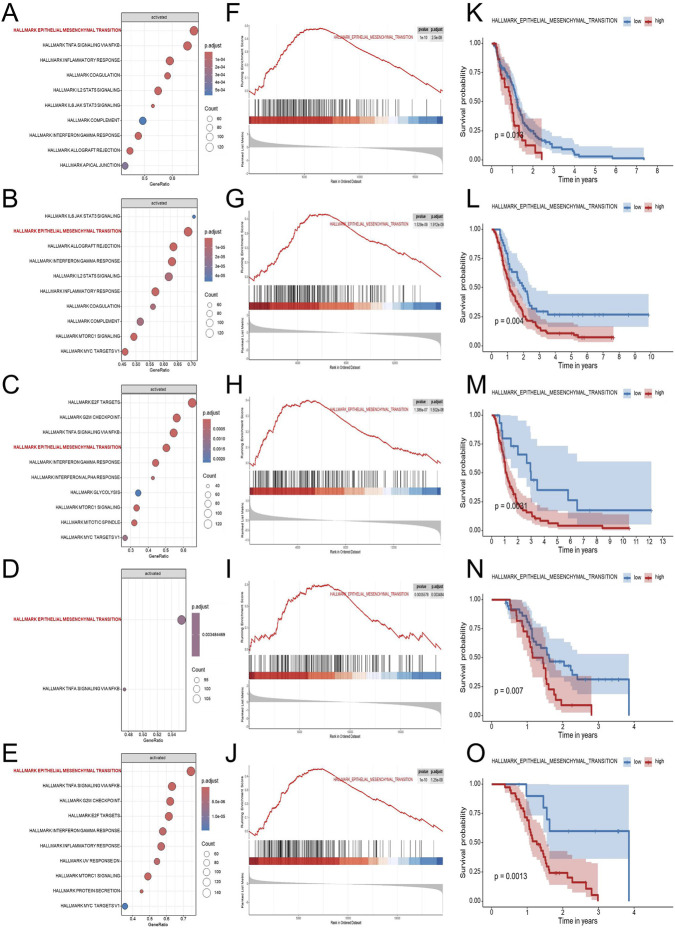
Identification and validation of EMT as a key prognostic pathway in GBM. To explore biological pathways associated with GBM prognosis, five independent transcriptomic datasets (TCGA-GBM, CGGA-325, CGGA-693, GSE74187, and GSE83300) were analyzed using Cox regression-based pathway enrichment analysis. Hallmark pathway enrichment analyses based on log_2_HR values from univariate Cox regression revealed EMT as one of the most significantly enriched pathways associated with poor survival across all five cohorts **(A–E)**. Gene set enrichment analysis demonstrated significant enrichment of the EMT pathway in the high-risk group, supporting its association with adverse clinical outcomes **(F–J)**. Kaplan-Meier survival analyses based on ssGSEA-derived EMT scores demonstrated that patients with high EMT activity exhibited significantly shorter overall survival compared with those with low EMT activity in each dataset **(K–O)**. Abbreviations: GBM, Glioblastoma multiforme; EDGs, EMT-driving genes; EMT, HALLMARK_EPITHELIAL_MESENCHYMAL_TRANSITION; log_2_HR, log_2_-transformed hazard ratio; GSEA, gene set enrichment analysis; NES, normalized enrichment score; ssGSEA, single-sample GSEA.

To explore genes associated with survival in GBM, we first performed univariate Cox regression analysis on mRNA transcripts in the TCGA-GBM cohort. The hazard ratios (HRs) for each gene was subsequently log_2_-transformed (log2HR) to better quantify its relative contribution to patient risk, allowing clearer distinction between risk-associated and protective genes. Genes were then ranked according to their transformed HR values, and this ranked gene list was subjected to GSEA to identify biological pathways enriched among genes conferring higher risk. As shown in Figure 2, GSEA revealed that genes associated with increased hazard were predominantly enriched in the HALLMARK_EPITHELIAL_MESENCHYMAL_TRANSITION (EMT) pathway. This result suggested that EMT-related biological processes are associated with adverse outcomes in GBM patients. To verify this finding, the same analysis was applied to four independent external GBM datasets (GSE83300, GSE74187, CGGA-693, and CGGA-325). The CGGA cohorts (derived largely from Chinese patients) provide paired transcriptomic and clinical annotation. Consistent with the TCGA, EMT was among the top enriched pathways across the validation cohorts, prompting us to further evaluate EMT activity at the sample level. We next quantified EMT pathway activity in each GBM sample using gene set variation analysis (GSVA) based on the hallmark EMT gene set. The resulting GSVA scores were used to represent the relative EMT activity of individual samples. Patients were subsequently stratified into high-EMT and low-EMT groups according to the GSVA score cutoff. Kaplan-Meier survival analysis revealed that patients with higher EMT scores exhibited significantly worse overall survival compared to those with lower scores. Importantly, this trend was consistently observed across all four external validation cohorts, further supporting the relevance of EMT pathway activation in GBM malignancy.

Encouraged by these findings, we next sought to systematically identify EMT-associated genes with potential prognostic relevance in GBM. To this end, genes that showed statistically significant associations with overall survival in univariate Cox regression analysis of the TCGA-GBM cohort were intersected with EMT-related genes obtained from the dbEMT2.0 (http://dbemt.bioinfo-minzhao.org/) and MSigDB databases. By intersecting these datasets, we identified a total of 145 EMT-driving genes (EDGs) for model development (Supplementary Table S1).

### Construction and validation of the EDGs signature

**FIGURE 3:**
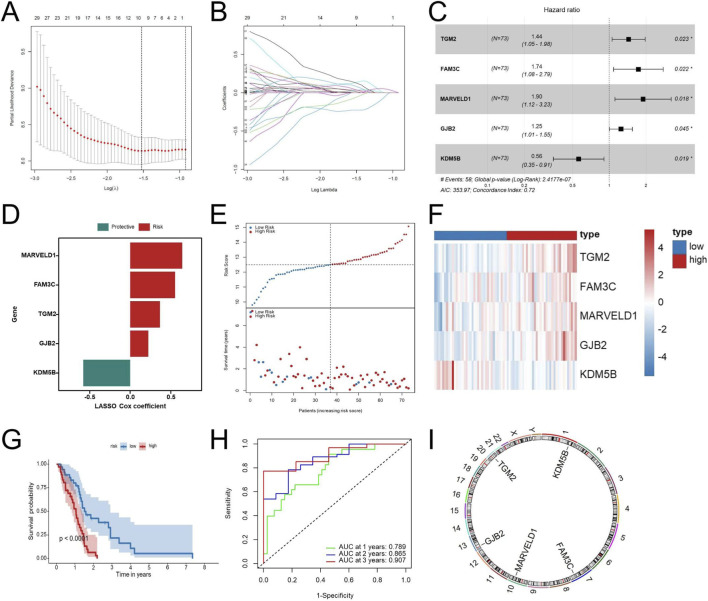
Construction of the EDGs signature. Ten-fold cross-validation curve of the LASSO Cox model showing partial likelihood deviance across λ values **(A)**. Coefficient trajectories of EMT-related genes across the λ sequence in the LASSO Cox regression **(B)**. Multivariate Cox regression analysis of the identified prognostic EDGs **(C)**. Risk coefficients of the prognostic EDGs **(D)**. Risk score distribution (top) and survival status of GBM patients (bottom) **(E)**. Heatmap displaying the expression profiles of the prognostic EDGs in high-risk and low-risk groups **(F)**. Kaplan-Meier survival analysis comparing OS between high-risk and low-risk patients **(G)**. ROC curve analysis for 1-year, 2-year, and 3-year OS **(H)**. Chromosomal locations of the five genes in the EDGs signature **(I)**. Risk groups were defined using the median risk score as the cutoff. Abbreviations: EDGs, EMT-driving genes; LASSO, least absolute shrinkage and selection operator; GBM, Glioblastoma multiforme; OS, overall survival; ROC, receiver operating characteristic; AUC, area under the curve. Statistical significance: *p < 0.05.

First, to construct and validate the prognostic model, the TCGA-GBM cohort was evenly split into a training cohort and an internal testing cohort. Additionally, four independent external validation cohorts, including two GEO datasets (GSE83300 and GSE74187) and two CGGA cohorts (CGGA-693 and CGGA-325) were used. Out of the 145 genes found to have significant associations with EMT following univariate Cox analysis, LASSO–Cox regression further narrowed the list to 9 candidates, and multivariate Cox regression finally identified 5 genes (TGM2, FAM3C, MARVELD1, GJB2, and KDM5B), which together formed the basis of the EMT-related risk score (Figures 3A–C). The risk score model was developed based on these five genes, and its calculation formula is as follows: Riskscore = 0.36681 × TGM2 + 0.55268 × FAM3C + 0.64165 × MARVELD1 + 0.22246 × GJB2 − 0.57815 × KDM5B (Figure 3D). Patients were stratified into high-risk and low-risk groups according to the median risk score. The risk score distribution, patient classification, and survival time for the two groups are illustrated in Figure 3E, while Figure 3F displays the expression patterns of five genes across the different risk groups. Figure 3G indicates that when patients were stratified into high- and low-risk categories, the high-risk group exhibited significantly shorter overall survival. Time-dependent ROC curve analysis was performed to further validate the robustness of the signature, resulting in AUC values of 0.789, 0.865, and 0.907 for 1-, 2-, and 3-year overall survival, respectively (Figure 3H). These results indicate strong discrimination in the TCGA internal cohorts. Figure 3I presents the chromosomal locations of the five genes.

**FIGURE 4:**
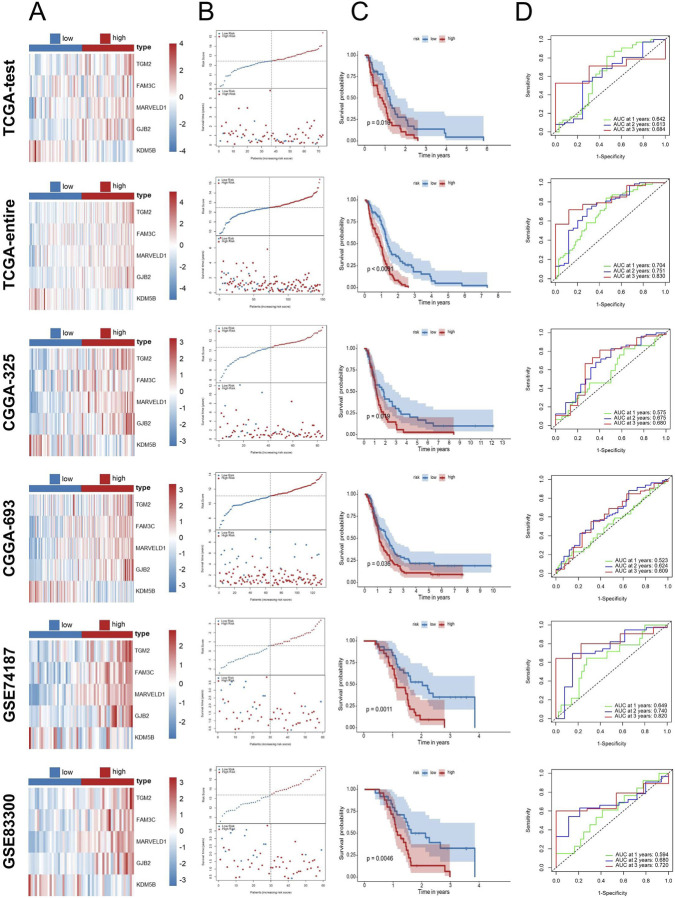
Validation of the EDGs signature. Validation of the EDGs signature in independent cohorts without data merging. Heatmap displaying the expression profiles of the prognostic EDGs between high-risk and low-risk groups in the TCGA-test, TCGA-entire, CGGA-325, CGGA-693, GSE74187, and GSE83300 cohorts **(A)**. Risk score distribution (top) and survival status (bottom) of GBM patients in the six cohorts **(B)**. Kaplan-Meier survival analysis comparing OS between high- and low-risk patients in the six cohorts **(C)**. ROC curve analysis for 1-year, 2-year, and 3-year OS in the six cohorts **(D)**. Abbreviations: EDGs, EMT-driving genes; GBM, Glioblastoma multiforme; TCGA, The Cancer Genome Atlas; CGGA, Chinese Glioma Genome Atlas; GEO, Gene expression omnibus; OS, overall survival; ROC, receiver operating characteristic; AUC, area under the curve.

To further evaluate the dependability and efficacy of the EDGs signature, survival analysis alongside ROC analysis was conducted in the TCGA test set and the full TCGA cohort as well as four external cohorts (GSE83300, GSE74187, CGGA-693, and CGGA-325). The risk score distribution and survival status among GBM patients are illustrated in Figures 4A,B. The Kaplan-Meier survival analysis (Figure 4C) demonstrated that, when evaluating both internal and external cohorts, individuals classified within the low-risk category displayed significantly improved survival rates in comparison to their high-risk counterparts, consistent with findings from the training cohort. Additionally, the ROC analysis revealed that the AUC values were consistently elevated across various cohorts, thereby supporting the robustness of this signature (Figure 4D).

### Association between EDGs signature and clinicopathological characteristics

**FIGURE 5:**
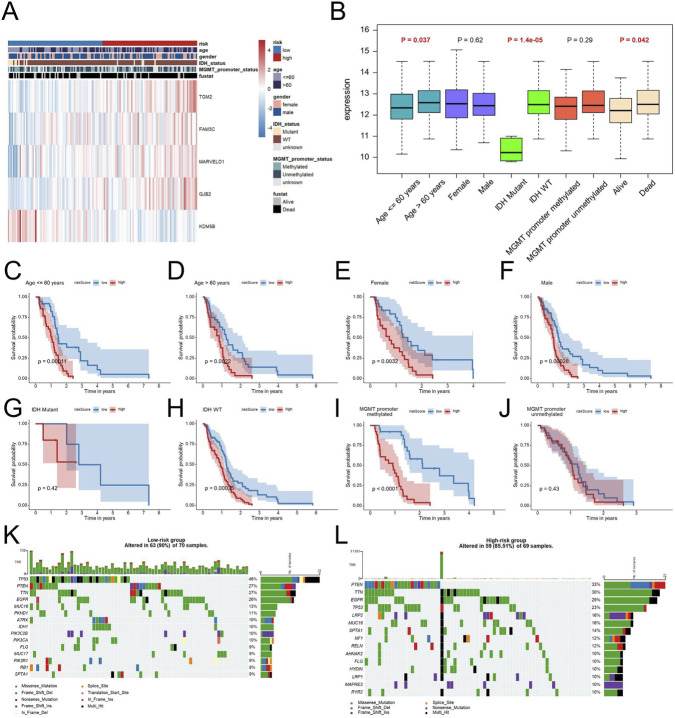
Clinical Relevance and Genomic Landscape of the EDGs Signature in GBM. Heatmap displaying the relationship between the EDGs signature and various clinicopathological features, including age, gender, IDH status, MGMT promoter status and survival status **(A)**. Box plots illustrating the distribution of risk scores across different clinical subgroups **(B)**. Kaplan-Meier survival analysis for patients stratified by age **(C,D)**, gender **(E,F)**, IDH status **(G,H)**, MGMT promoter status **(I,J)**. Mutation landscape of the low-risk **(K)** and high-risk **(L)** groups, showing the frequency and distribution of somatic mutations in GBM patients. Mutation profiles are shown to illustrate differences in genomic alteration patterns between risk groups. Abbreviations: EDGs, EMT-driving genes. TMB, tumor mutation burden.

In order to evaluate the clinical significance of the EDGs signature, we conducted an extensive correlation analysis with various clinicopathological features. The results, illustrated in heatmaps and box plots (Figures 5A,B), demonstrated notable associations between the EDGs signature and diverse clinicopathological characteristics. Specifically, it was found that patients aged ≥60 years, those with IDH mutation, and individuals who had succumbed to their illness presented with elevated risk scores. Additionally, the stratified analysis indicated that within distinct subgroups categorized by age, sex, IDH status, and MGMT promoter methylation, patients classified as high-risk consistently displayed inferior OS outcomes (Figures 5C–J). Importantly, the prognostic relevance of this signature remained robust across various clinical subgroups, thereby reinforcing its credibility.

Analysis of genomic alterations revealed that Overall mutation frequency was in the low-risk cohort was markedly higher in comparison to that in the high-risk cohort (90% vs. 85.51%) (Figures 5K,L).

### Nomogram construction

**FIGURE 6:**
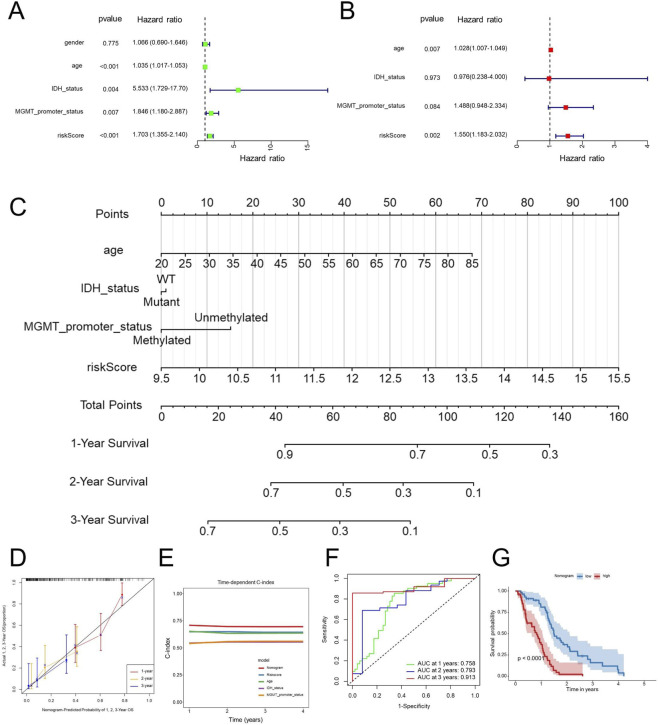
Nomogram construction and validation. Univariate **(A)** and multivariate **(B)** Cox regression analyses of prognostic factors, including clinical and the EDGs signature. Nomogram incorporating significant prognostic factors for predicting 1-year, 2-year, and 3-year OS **(C)**. Calibration curves assessing the predictive accuracy of the nomogram for 1-year, 2-year, and 3-year OS **(D)**. Time-dependent C-index comparison between the nomogram and individual prognostic factors **(E)**. ROC curve analysis for 1-year, 2-year, and 3-year OS predictions using the nomogram **(F)**. Kaplan-Meier survival analysis based on nomogram-derived risk stratification **(G)**. Abbreviations: OS, overall survival; Concordance index, C-index; GBM, Glioblastoma multiforme; ROC, receiver operating characteristic; AUC, area under the curve.

In order to evaluate whether our identified signature functions as an independent prognostic indicator for patients diagnosed with GBM, we performed both univariate and multivariate Cox regression analyses. The findings revealed that both the risk score and patient age served as independent prognostic factors (Figures 6A,B), suggesting that the risk score remained independently associated with the prognosis of GBM of other clinical parameters. To improve the clinical relevance of our research, we developed a nomogram model that integrates key prognostic variables, including age, IDH status, MGMT promoter status, and the risk score (Figure 6C).

The developed nomogram serves as an interpretable tool for predicting overall survival in patients diagnosed with GBM, thus improving the clinical utility of the associated risk score. The results revealed that the nomogram exhibited strong predictive accuracy for overall survival at the 1-year, 2-year, and 3-year marks in GBM patients (Figure 6D). Notably, the C-index for the nomogram showed a substantial enhancement when compared to individual predictors (Figure 6E), emphasizing the benefits of utilizing a combined model. Additionally, the ROC AUC values for predicting overall survival at 1 year, 2 years, and 3 years using the nomogram were recorded at 0.758, 0.793, and 0.913, respectively (Figure 6F), further reinforcing the efficacy and clinical significance of this prognostic nomogram. Consistently, according to the Kaplan-Meier survival curves, patients classified with higher nomogram scores experienced significantly poorer OS compared with those with lower scores (Figure 6G).

### WGCNA and GSEA

**FIGURE 7:**
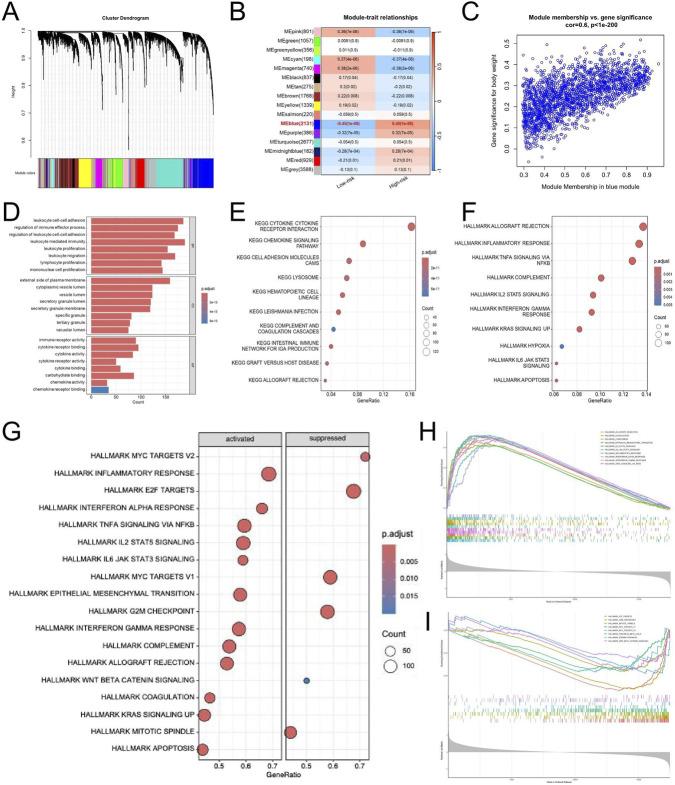
WGCNA and Pathway Enrichment Analyses of the EDGs Signature. WGCNA was performed using standard parameters to identify co-expression modules associated with risk groups **(A)**. Module-trait relationship heatmap showing the correlation between gene modules and risk groups **(B)**. Scatter plot illustrating the correlation between gene significance and module membership in the blue module **(C)**. GO enrichment analysis of genes in the blue module **(D)**. KEGG pathway enrichment analysis of genes in the blue module **(E)**. Hallmark pathway enrichment analysis of genes in the blue module **(F)**. Bubble plot showing hallmark pathways significantly enriched in the high-risk and low-risk groups based on GSEA **(G)**. Enrichment plots of key hallmark pathways activated in the high-risk group **(H)**. Enrichment plots of immune-related hallmark pathways suppressed in the high-risk group **(I)**. Abbreviations: WGCNA, weighted gene co-expression network analysis; EDGs, EMT-driving genes; GO, gene ontology; KEGG, Kyoto encyclopedia of genes and genomes. GSEA, gene set enrichment analysis.

To investigate the gene modules related to the EDGs signature, we conducted a weighted gene co-expression network analysis (WGCNA). Within the TCGA-GBM dataset, a soft-thresholding power of β = 12 was utilized, resulting in the identification of 12 unique gene modules through the application of the topological overlap matrix (TOM) (Figure 7A). Notably, the blue module demonstrated the most significant association with the risk score (Figure 7B). Subsequent analyses indicated that the blue module exhibited the highest positive correlation with the risk score, yielding a correlation coefficient of 0.6 and P < 1 × 10^−200^ (Figure 7C). We conducted enrichment analyses for Gene Ontology (GO) and the Kyoto Encyclopedia of Genes and Genomes (KEGG) pathways on genes associated with the blue module. The pathways were ranked by P-value to identify the most significantly enriched biological functions and signaling processes. Functional annotation showed that genes in the blue module were predominantly linked to immune-related processes. GO analysis highlighted enrichment in leukocyte-mediated immunity, immune receptor activity, and components of the plasma membrane (Figure 7D), while KEGG pathways mainly involved cytokine interactions, chemokine signaling, and CAMs (Figure 7E). In line with these findings, Hallmark analysis revealed activation of immune processes such as inflammatory and interferon-γ responses, allograft rejection, complement activation, and IL2-STAT5 and IL6-JAK-STAT3 signaling (Figure 7F). Furthermore, GSEA was conducted to investigate the potential biological differences between the high- and low-risk groups defined by the EMT-related prognostic signature. The analysis revealed that the high-risk group was markedly enriched in immune and inflammation-related pathways, including inflammatory response, IL6-JAK-STAT3 signaling, IL2-STAT5 signaling, TNFα signaling via NF-κB, interferon-alpha and gamma responses, and epithelial-mesenchymal transition (Figures 7G,H). Conversely, the low-risk group showed enrichment in pathways related to apoptosis, KRAS signaling, WNT/β-catenin signaling, coagulation, pancreatic beta cell function, and spermatogenesis (Figure 7I). These findings suggest that high-risk tumors are characterized by enhanced immune activation and inflammatory signaling, whereas low-risk tumors exhibit greater enrichment of metabolic and differentiation-related processes, reflecting distinct biological behaviors associated with EMT-mediated malignancy.

### Immune infiltration analysis

**FIGURE 8:**
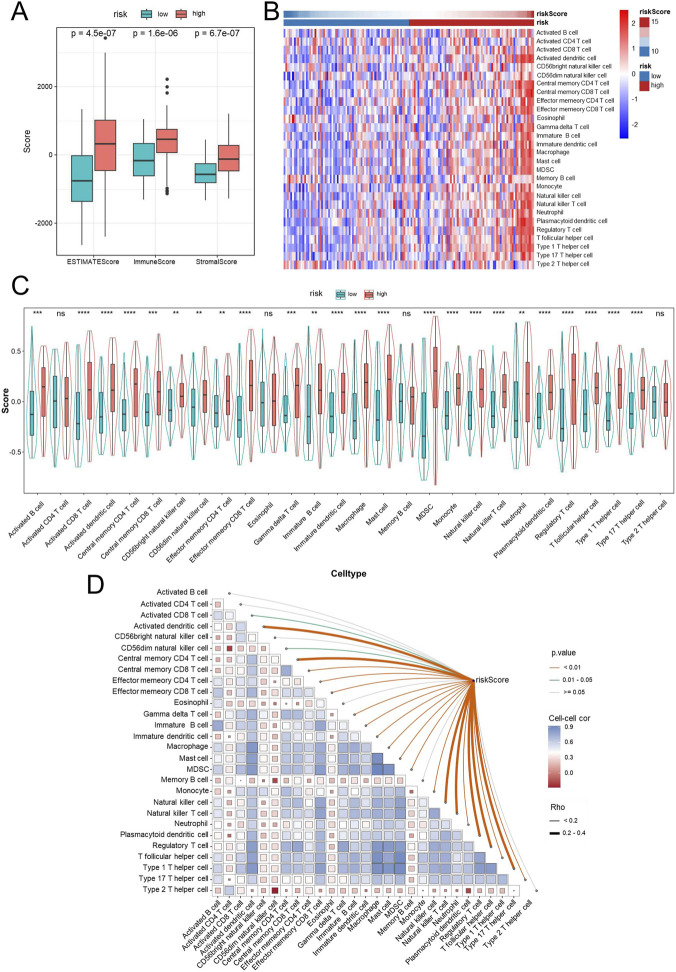
Immune infiltration analysis in high- and low-risk GBM patients. Immune infiltration was estimated using ssGSEA. Box plots comparing ESTIMATE score, immune score, and stromal score between high- and low-risk groups **(A)**. Heatmap displaying the infiltration levels of tumor-infiltrating immune cells in high- and low-risk groups based on ssGSEA **(B)**. Violin plots showing the distribution of immune cell infiltration levels between high- and low-risk groups **(C)**. Correlation between risk scores and immune cell infiltration **(D)**. Abbreviations: GBM, Glioblastoma multiforme; ssGSEA, single-sample gene set enrichment analysis. Statistical significance: **p < 0.01, ***p < 0.001, ****p < 0.0001, ns: not significant.

Evaluation of the tumor immune microenvironment revealed a distinctive immune activation profile in the high-risk group, with significantly higher immune, stromal, and ESTIMATE scores compared with the low-risk group (Figure 8A). Consistently, infiltration of diverse immune cell subsets was elevated in these patients, suggesting that immune infiltration was higher in the high-risk group, consistent with the observed adverse survival (Figure 8B). To further dissect the immune composition, ssGSEA was applied to quantify the infiltration of 28 immune cell subsets. As shown in Figure 8C, the high-risk group exhibited widespread elevation across both effector and immunosuppressive populations. Enrichment of activated CD8^+^ and memory CD4^+^ T cells, NK cells, and dendritic cells was observed in the high-risk group, suggesting an intensified immune response within the tumor microenvironment. Meanwhile, the proportions of regulatory T cells (Tregs) and myeloid-derived suppressor cells (MDSCs) were also increased, reflecting the coexistence of suppressive immune components. These results collectively suggest that high-risk tumors possess an “immune-inflamed yet immunosuppressed” microenvironment, characterized by abundant immune infiltration yet potentially impaired antitumor immune efficacy. Additional correlation analysis showed a positive association between the risk score and multiple immune cell types (Figure 8D). Key cell types, including Activated CD4 T cell, Activated CD8 T cell, activated dendritic cell, Central memory CD8 T cell, Macrophage, and Natural killer T cell, showed positive correlations with risk score, as indicated in the heatmap. Immature B cell, Mast cell, and Plasmacytoid dendritic cell exhibited particularly strong associations, while only a few cell types showed weak or non-significant correlations. These results suggest that higher risk scores may correlate with increased infiltration or activation of these immune cells, reflecting a specific immune microenvironment feature.

### Drug sensitivity analysis

**FIGURE 9:**
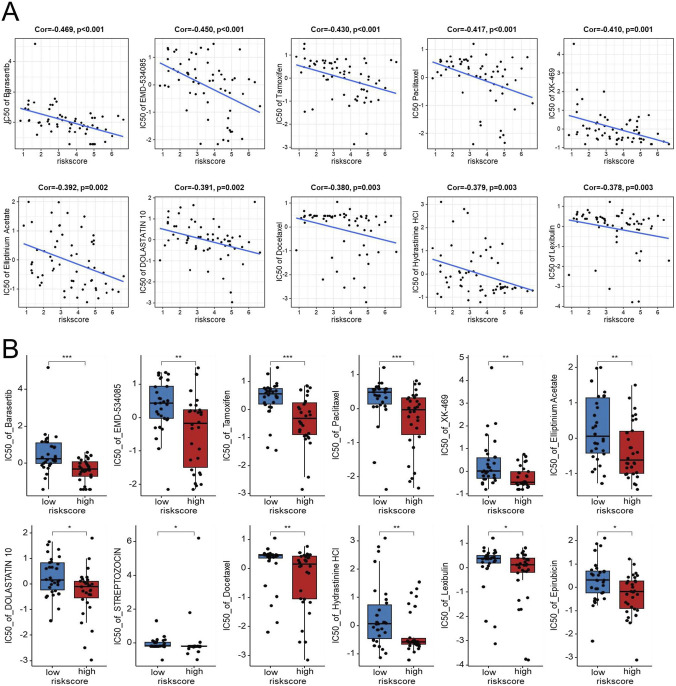
Exploratory analysis of the association between the EDGs signature and drug sensitivity in the NCI-60 cell line panel. Scatter plots showing the negative correlation between the risk score and IC50 values for several drugs, including Barasertib, EMD-534085, Tamoxifen, Paclitaxel, XK-469, Elliptinium Acetate, DOLASTATIN 10, Docetaxel, Hydrastinine HCl, and Lexibulin **(A)**. Boxplots comparing IC50 values for the above drugs, along with the added Streptozocin and Epirubicin Acetate, between high-risk and low-risk groups **(B)**. Abbreviations: EDGs, EMT-driving genes. Statistical significance: *p < 0.05, **p < 0.01, ***p < 0.001, IC50 half maximal inhibitory concentration.

To nominate candidate agents for GBM patients with high EMT-related risk scores, we analyzed the correlation between the risk score and drug sensitivity using the CellMiner database. Correlation analysis revealed that the EMT-based risk score was significantly negatively correlated with the IC50 values of multiple compounds, indicating that high-risk samples were more sensitive to these drugs. As shown in Figures 9A,B, the IC50 values of Barasertib, EMD-534085, Tamoxifen, Paclitaxel, XK-469, Elliptinium Acetate, DOLASTATIN 10, STREPTOZOCIN, Docetaxel, Hydrastinine HCl, Lexibulin, and Epirubicin were markedly lower in the high-risk group than in the low-risk group. These findings suggest that patients classified as high risk according to the EMT signature may be more responsive to these agents, highlighting potential therapeutic opportunities for this aggressive GBM subtype.

### Identification of GJB2 as the most important gene in EDGs signature

**FIGURE 10:**
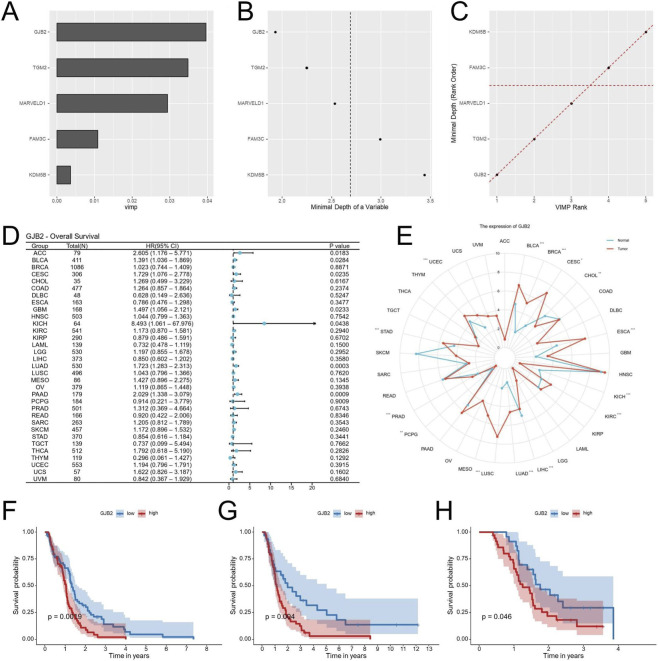
Identification of GJB2 as the key gene in the EDGs signature. Random Forest analysis ranking the importance of the five EDGs, identifying GJB2 as the most pivotal gene **(A–C)**. Univariate Cox regression analysis of GJB2 expression and overall survival across TCGA cancers **(D)**. Pan-cancer analysis of GJB2 expression across multiple tumor types in the TCGA database **(E)**. Kaplan-Meier survival analysis showing the association between GJB2 expression and OS in the TCGA-GBM **(F)**, GSE74187 **(G)**, and CGGA-325 **(H)**. Abbreviations: EDGs, EMT-driving genes; OS, overall survival; TCGA, The Cancer Genome Atlas; CGGA, Chinese Glioma Genome Atlas; GEO, Gene expression omnibus; Statistical significance: *p < 0.05, **p < 0.01, ***p < 0.001, ns: not significant.

To pinpoint the most influential genes within the EDGs signature, a Random Forest analysis was conducted. Among the five EDGs, GJB2 emerged as the top-ranked gene, suggesting its pivotal contribution to the prognostic model (Figures 10A–C). Subsequent Pan-cancer survival analyses (univariate Cox and Kaplan-Meier) were performed to assess the prognostic impact of GJB2 expression. Analysis of pan-cancer cohorts indicated that high GJB2 expression was significantly associated with unfavorable overall survival in several malignancies, including ACC, BLCA, CESC, GBM, KICH, LUAD, and PAAD (Figure 10D). Besides, data from the TCGA database revealed that GJB2 expression was expressed at higher levels in multiple tumor types compared with adjacent normal tissues (Figure 10E). Kaplan-Meier survival analysis was performed across five independent GBM cohorts (TCGA-GBM, GSE83300, GSE74187, CGGA-693 and CGGA-325) to evaluate the prognostic value of GJB2 expression. Consistently, patients with high GJB2 expression tended to exhibit shorter overall survival across all datasets, with statistically significant differences observed in the TCGA, GSE74187, and CGGA-325 cohorts (Figures 10F-H). Altogether, these data position GJB2 as an important molecular driver of GBM progression and a viable candidate for therapeutic development.

### GJB2 knockdown inhibits the proliferation and migration of GBM cells

**FIGURE 11:**
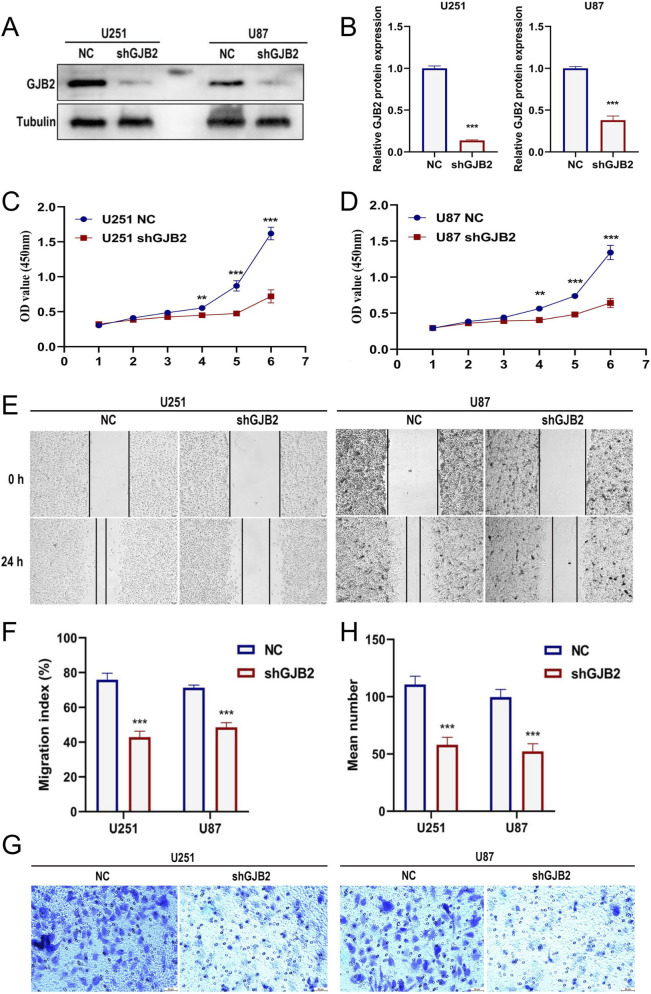
Functional effects of GJB2 knockdown in GBM cells.qRT-PCR analysis confirming the knockdown efficiency of shGJB2 in U251 and U87 cells **(A-B)**. CCK-8 assay evaluating the proliferation of U251 **(C)** and U87 **(D)** cells after GJB2 knockdown. Scratch wound healing assay measuring the migration rate of U251 and U87 cells after GJB2 knockdown **(E)**, with quantification shown in **(F)**. Transwell migration assay showing reduced migratory ability of U251 and U87 cells after GJB2 knockdown **(G)**, with quantification of migrated cells shown in **(H)**. Uncropped Western blot images are provided in Supplementary Figure S3. Abbreviations: GBM, Glioblastoma multiforme; qRT-PCR, quantitative real-time polymerase chain reaction; CCK-8, cell counting kit-8; shRNA, short hairpin RNA. Statistical significance: *p < 0.05, **p < 0.01, ***p < 0.001, ns: not significant.

To elucidate the functional role of GJB2, GBM cell lines (U251 and U87) were transfected with shGJB2 lentivirus, leading to efficient gene silencing (Figures 11A,B). CCK-8 assays revealed that GJB2 silencing significantly inhibited cell proliferation (Figures 11C,D). Additionally, the scratch wound assay revealed that reducing GJB2 expression considerably inhibited migration of GBM cells (Figures 11E,F). Furthermore, Transwell invasion assays demonstrated a significant decrease in the migratory capacity of GBM cells subsequent to the knockdown of GJB2 when contrasted with the control cells (Figures 11G,H). In summary, these findings suggest that the silencing of GJB2 leads to suppressed proliferation, migration, and invasion of GBM cells *in vitro*, thereby underscoring its potential function as an oncogenic driver and a viable therapeutic target in the progression of GBM.

## Discussion

Recent advancements in GBM biology have underscored its pronounced molecular and cellular heterogeneity, which continues to complicate effective clinical management (Yabo et al., 2022). Despite progress in multimodal therapies, including surgical resection, radiotherapy, and temozolomide-based chemotherapy, GBM remains lethal due to profound therapeutic resistance and cellular plasticity (Amirmahani et al., 2025; Pu et al., 2025). Among the molecular programs driving this aggressiveness, EMT—often described in glioblastoma as an EMT-like or proneural-to-mesenchymal transition (PMT) program—has emerged as a central determinant of invasion, therapeutic resistance, and recurrence (Kim et al., 2021). However, translating these insights into clinically applicable prognostic tools remains a key challenge.

In this study, we first performed survival analysis to explore biological pathways associated with GBM prognosis. Although EMT-related prognostic signatures have been primarily studied in cancers such as lung adenocarcinoma (Zhou et al., 2022), we sought to investigate their relevance in GBM. Using univariate Cox regression in the TCGA cohort, genes were ranked by hazard ratio and analyzed by GSEA, which identified EMT as a leading pathway associated with poor prognosis. This finding was consistently replicated across independent CGGA and GEO datasets, confirming the robustness of EMT as a key prognostic process in GBM. A key strength was the pathway-first strategy: we anchored model development to a defined biological program before feature selection. Guided by this discovery, we compiled a set of 145 EDGs and constructed an EMT-based risk score. Integration of this score with age, IDH status, and MGMT promoter methylation yielded a clinically integrated nomogram, which demonstrated superior predictive accuracy compared with previous gene-only models that lacked clinical variables (Luo et al., 2024; Xue et al., 2024).

The EMT-based signature demonstrated strong and independent predictive power for overall survival. Distinct survival stratification was evident between high- and low-risk groups (P < 0.0001), and both univariate and multivariate Cox regression analyses verified that the risk score served as an independent prognostic indicator. Time-dependent ROC analysis showed AUC values of 0.788, 0.793, and 0.813 for 1-, 2-, and 3-year survival, respectively, and calibration curves demonstrated good agreement between predicted and observed survival rates. Notably, the integrated nomogram achieved higher predictive performance than models based solely on gene signatures or clinical factors, supporting its translational potential for individualized prognosis and risk-adapted management in GBM patients.

Beyond its prognostic strength, our EMT-related signature provides biological insight into GBM aggressiveness. Mutation analysis indicated that TP53 and IDH1 mutations were enriched in the low-risk group, whereas high-risk tumors harbored fewer classical driver alterations, suggesting that EMT-associated risk is driven primarily by transcriptional and microenvironmental programs. Genes most strongly correlated with the riskscore were enriched in cytokine signaling, leukocyte adhesion, and immune regulation pathways, suggesting that EMT-associated risk in GBM aligns within an inflammatory transcriptional context. This aligns with reports that mesenchymal transformation in GBM is driven by a core inflammatory transcriptional network involving factors such as STAT3 and C/EBPβ, and reinforced by cytokines such as IL-6 (Carro et al., 2010). GSEA further revealed enrichment of hallmark EMT, TGF-β, hypoxia, and NF-κB/IL6-STAT3 signaling—pathways known to sustain mesenchymal plasticity, stem-like features, and immunosuppressive remodeling (Lamano et al., 2019; Liu et al., 2021).

Our immune microenvironment analysis revealed that high-risk GBM tumors exhibited significantly higher ImmuneScore and StromalScore, along with widespread immune cell infiltration, yet these patients experienced poorer overall survival. Notably, this pattern is consistent with the predominance of immunosuppressive cell populations in GBM. Tumor-associated macrophages (TAMs) and microglia, which can constitute a substantial fraction of the tumor mass, often acquire an immunosuppressive (frequently M2-like) phenotype that supports tumor growth and suppresses cytotoxic T-cell activity through IL-10 and TGF-β secretion (Dziurzynski et al., 2011). Similar observations have been reported in GBM, where tumors rich in immune and stromal components often display dysfunctional immune activation and poor prognosis (Wang et al., 2017; Chen and Hambardzumyan, 2018). Importantly, although CD8^+^ T cells are often detectable in GBM tissues, accumulating evidence indicates that they commonly exhibit an exhausted and functionally impaired phenotype rather than effective cytotoxic activity. Transcriptomic studies have shown that glioblastoma harbors some of the most pronounced T-cell exhaustion signatures among solid tumors, characterized by inhibitory receptor expression and reduced effector function (Woroniecka et al., 2018). In addition, systemic immune dysregulation in intracranial tumors, including sequestration of T cells in the bone marrow, further limits effective antitumor immunity despite apparent immune infiltration (Chongsathidkiet et al., 2018). Consistent with these observations, cytokine-driven signaling pathways such as IL6-JAK-STAT3 and TGF-β signaling—both enriched in our high-risk group—have been implicated in promoting macrophage polarization and T-cell dysfunction in glioblastoma (Liu et al., 2022; Hourani et al., 2025). Collectively, these findings support the concept of an “immune-inflamed yet immunosuppressed” tumor microenvironment in high-risk GBM, in which immune cell infiltration fails to translate into effective antitumor immunity due to exhaustion and suppression mechanisms (Verhaak et al., 2010; Hambardzumyan et al., 2016), thereby motivating exploration of candidate drug sensitivities associated with this risk state.

Correlation analysis with the CellMiner database revealed that higher risk scores were positively associated with increased sensitivity to several compounds, including paclitaxel and tamoxifen, both of which have shown antitumor activity in glioma models. Paclitaxel, a microtubule-stabilizing agent, has been reported to induce apoptosis and inhibit invasion in GBM cells, particularly when delivered via nanoparticle or convection-enhanced systems that improve brain penetration (Lidar et al., 2004; AbdEl-Haq et al., 2023). Similarly, Tamoxifen, a selective estrogen receptor modulator, exhibits antiproliferative and pro-apoptotic effects independent of estrogen signaling, and its efficacy can be enhanced through modulation of the PI3K/AKT pathway (Li et al., 2011). Clinical studies have also indicated that subsets of patients with malignant glioma may achieve disease stabilization with high-dose Tamoxifen therapy (Couldwell et al., 1996). It should be noted that these drug–risk score associations were derived from pan-cancer cell line data and should be interpreted as exploratory. Validation in GBM-specific cellular or patient-derived models will be required to establish biological and therapeutic relevance. Collectively, these findings imply that EMT activation influences therapeutic sensitivity in GBM, leading us to focus on key molecular drivers underlying this phenotype and identify GJB2 as a pivotal component of our prognostic model.

Random Forest analysis identified GJB2 (Connexin-26) as the most influential gene within the signature. GJB2 encodes a gap junction protein mediating intercellular communication and coordinating oncogenic processes such as proliferation, migration, and stress adaptation (Naus and Laird, 2010; Aasen et al., 2019). In gliomas, connexin-43 (Cx43, encoded by GJA1) has been shown to promote invasion and resistance through heterocellular communication with astrocytes, facilitating calcium and metabolic signaling (Sin et al., 2016; Khosla et al., 2020). While the tumor-promoting role of Cx43 is established, the function of Cx26 in GBM remains underexplored. Functional assays in our study revealed that GJB2 knockdown suppressed GBM cell proliferation and migration. These findings provide functional evidence that GJB2 in promoting aggressive cellular phenotypes in GBM, potentially by modulating intercellular communication pathways that support tumor plasticity. Although its molecular mechanisms remain to be clarified, prior studies suggest connexins can modulate calcium flux, PI3K/AKT, and STAT3 signaling, linking gap junction communication with mesenchymal transition and immune regulation (Trosko and Ruch, 2002). In addition, transcriptomic analyses revealed a consistent positive association between GJB2 expression and EMT-related signatures, supporting a potential link between GJB2 and EMT programs, although direct mechanistic validation remains to be explored. Collectively, GJB2 emerges as a potential oncogenic driver bridging EMT-associated transcriptional programs and tumor invasiveness.

Despite these promising findings, several limitations should be acknowledged. First, the prognostic signature was derived from retrospective transcriptomic datasets from TCGA, CGGA, and GEO, which inherently differ in patient composition and experimental platforms. Each cohort was processed and analyzed independently without cross-cohort data integration; therefore, conventional batch-effect correction and cross-platform normalization were not applied. Moreover, the model was trained on RNA-seq data and validated across both RNA-seq and microarray cohorts, and intrinsic differences in expression scale and dynamic range between platforms may contribute to variability in effect size across datasets. Nevertheless, the directionality of prognostic associations remained consistent across all cohorts, supporting the biological robustness of the EMT-based signature. Despite the multi-cohort retrospective validation performed in this study, further validation in independent prospective cohorts is warranted. We are currently collecting additional glioblastoma samples and preparing transcriptomic sequencing to enable future experimental validation of the EMT-based prognostic signature. Second, although functional assays verified that GJB2 enhances GBM proliferation and migration *in vitro*, further *in vivo* studies are necessary to define its role in tumor progression and treatment response. Third, while the EMT signature is closely linked to immune and inflammatory pathways, the underlying mechanisms connecting GJB2 with EMT activation and immune remodeling remain unclear. Future studies should explore the downstream signaling networks and interaction partners of GJB2 within the EMT regulatory axis.

In summary, this study establishes a robust, biologically informed prognostic signature integrating EMT-related gene expression with clinical parameters for GBM. The identification and functional validation of GJB2 provide a mechanistic anchor for the model, linking intercellular communication to mesenchymal activation and tumor aggressiveness. These findings offer new insights into EMT-driven plasticity and immune modulation in GBM, presenting potential avenues for precision-targeted therapeutic strategies.

## Conclusion

In conclusion, this study systematically developed an EMT-related gene signature in GBM using a GSEA-guided strategy combined with Cox regression analyses across multiple cohorts. Incorporating key clinical variables into a composite nomogram substantially improved prognostic discrimination, enabling stratification of GBM patients into distinct risk groups. Furthermore, GJB2 was identified as a key gene within the signature, exerting a critical influence on tumor progression. Functional assays revealed that GJB2 promotes glioma cell proliferation and migration *in vitro*, supporting a pro-tumorigenic role and suggesting that GJB2 may serve as a prognostic marker and therapeutic target. Collectively, these findings highlight insights into the association between EMT-driven plasticity and immune remodeling in glioblastoma, supporting more precise prognostic assessment and the development of risk-informed therapeutic strategies in this challenging disease.

## Data Availability

The original contributions presented in the study are included in the article/Supplementary Material, further inquiries can be directed to the corresponding authors.
