# Supplementary figures and images for: Integrative analysis of EMT-driving genes identifies a prognostic signature and GJB2 as a potential biomarker in glioblastoma
Source: Front Cell Dev Biol. 2026 Jan 14;13:1754988. doi: 10.3389/fcell.2025.1754988 (PMC12847059; doi:10.3389/fcell.2025.1754988)

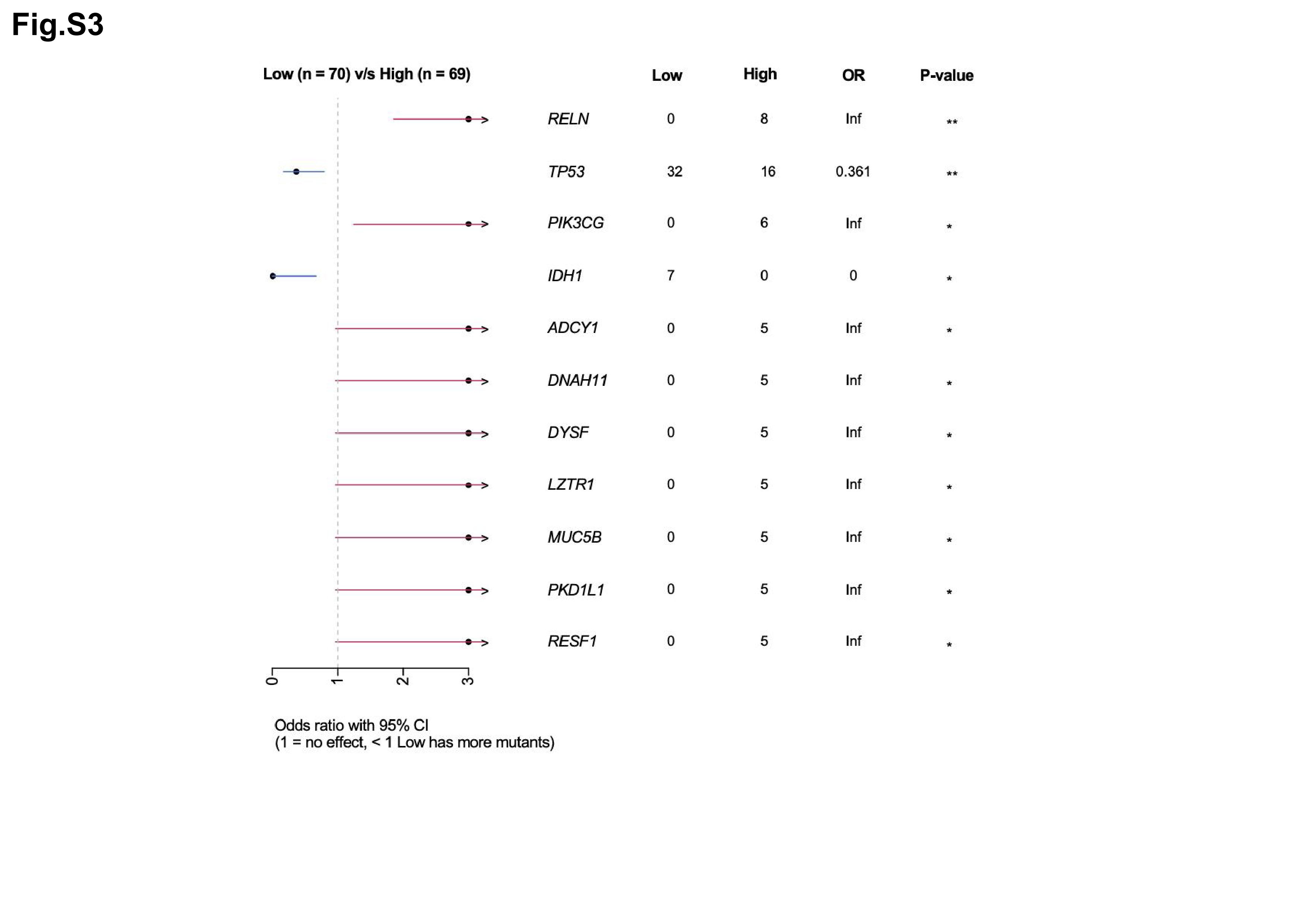

Supplement: Supplementary file 1 [file Image3.jpeg]

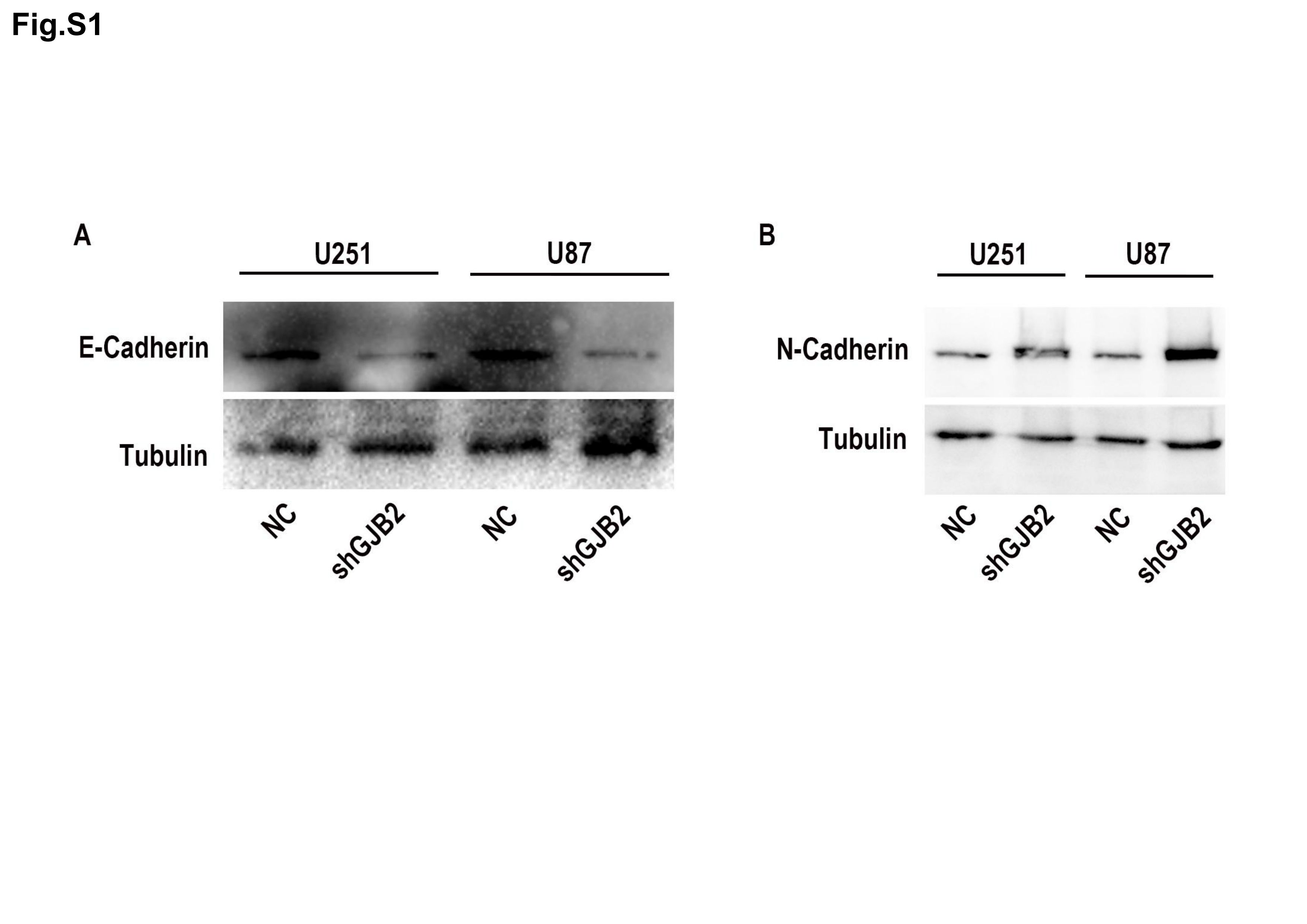

Supplement: Supplementary file 2 [file Image1.jpeg]

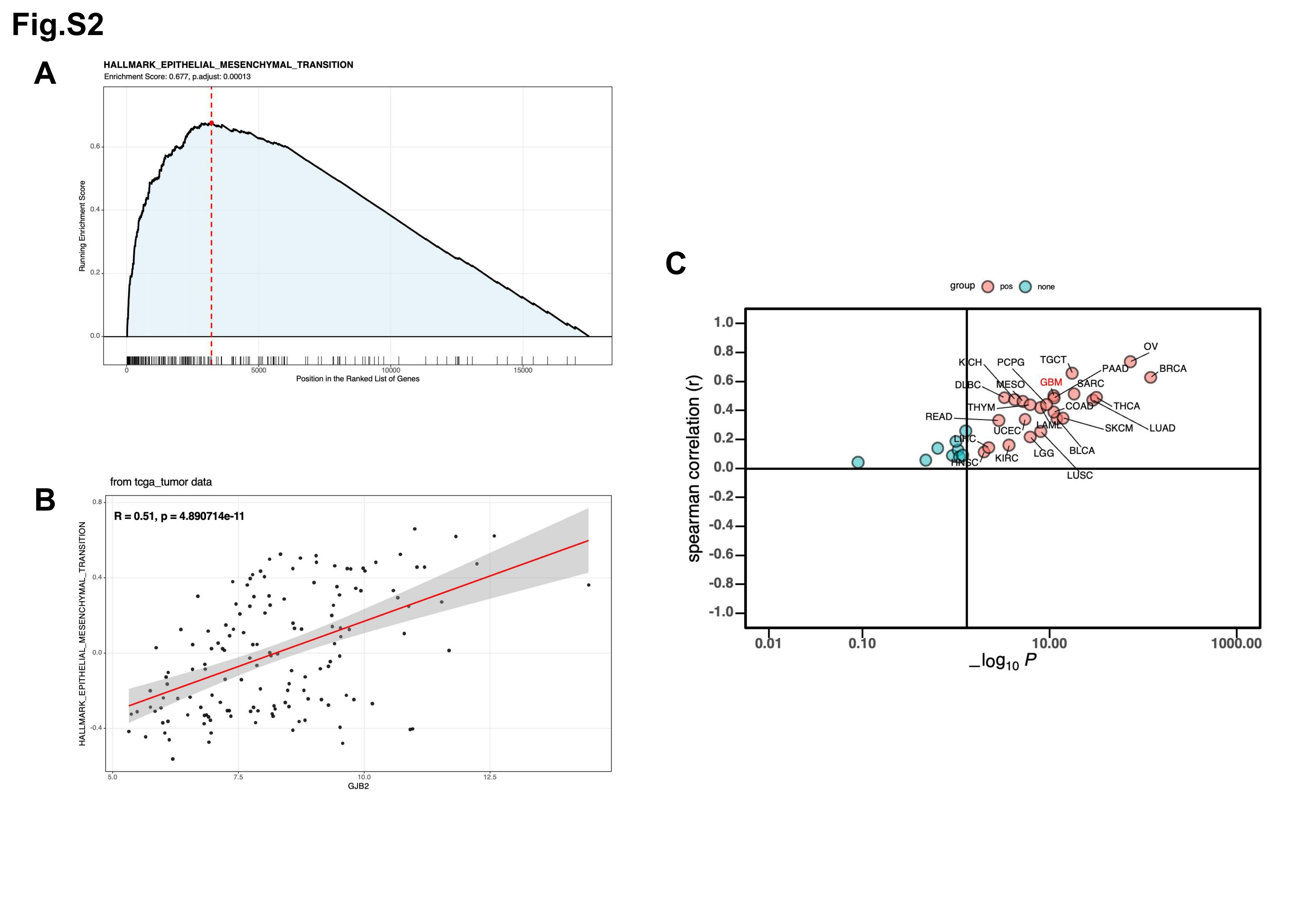

Supplement: Supplementary file 3 [file Image2.jpeg]
